# Machine learning predictors of cardiovascular autonomic neuropathy in type 2 diabetes: insights from a prospectively collected single-center registry

**DOI:** 10.3389/fendo.2026.1965013

**Published:** 2026-10-15

**Authors:** Katalin Farkas, Viktor Vedelek, Máté Vámos, Éva Csajbók, István Baczkó, János Zádori, Andrea Szentesi, Péter Hegyi, Tamás Várkonyi, Csaba Lengyel, Anna Vágvölgyi

**Affiliations:** 1 Department of Medicine, Albert Szent-Györgyi Medical School, University of Szeged, Szeged, Hungary; 2 Department of Genetics, Faculty of Science and Informatics, University of Szeged, Szeged, Hungary; 3 Department of Pharmacology and Pharmacotherapy, Albert Szent-Györgyi Medical School, University of Szeged, Szeged, Hungary; 4 Center of Reproductive Medicine, Department of Obstetrics and Gynecology, Albert Szent-Györgyi Medical School, University of Szeged, Szeged, Hungary; 5 Institute for Translational Medicine, Medical School, University of Pécs, Pécs, Hungary; 6 Centre for Translational Medicine, Semmelweis University, Budapest, Hungary; 7 Institute of Pancreatic Diseases, Semmelweis University, Budapest, Hungary; 8 Translational Pancreatology Research Group, Interdisciplinary Centre of Excellence for Research Development and Innovation, University of Szeged, Szeged, Hungary

**Keywords:** autonomic score, cardiovascular autonomic neuropathy, diabetes duration, machine learning, T2DM

## Abstract

**Background:**

Cardiac autonomic neuropathy (CAN) is a severe complication of type 2 diabetes (T2DM) associated with increased cardiovascular morbidity and mortality. Machine learning (ML) can integrate multidimensional clinical data and identify features associated with CAN. We aimed to characterize predictors of CAN categories using ML.

**Methods:**

Data were obtained from a prospective single-center observational registry of patients with T2DM undergoing standardized cardiovascular autonomic reflex tests (CARTs) based on Ewing’s protocol. Demographic, anthropometric, clinical, laboratory, and medication data were analyzed. Patients were classified as CAN0 (no or borderline CAN), CAN1 (early CAN), or CAN2 (definite or severe CAN). Three extreme gradient boosting models were evaluated: a CART-inclusive model, a CART-excluded model restricted to patients with T2DM, and a CART-excluded model supplemented with non-diabetic controls without abnormal CART results. Model performance was assessed using area under the receiver operating characteristic curve (AUC), and feature contributions were interpreted using SHapley Additive exPlanations values.

**Results:**

Among 693 patients with T2DM, 19 (2.7%) had completely normal autonomic function, whereas 674 (97.3%) exhibited at least one borderline or abnormal CART result. Greater CAN severity was associated with older age, longer diabetes duration, higher systolic blood pressure, and poorer renal function. The CART-inclusive model achieved perfect discrimination in the best iteration (weighted AUC, 1.00; accuracy, 100%), with all five CARTs ranking among the leading predictors. After CART exclusion, performance declined markedly (weighted AUC, 0.64), with class-specific AUCs of 0.59 for CAN0, 0.59 for CAN1, and 0.68 for CAN2. Inclusion of non-diabetic controls improved the weighted AUC to 0.78, with the greatest improvement for CAN0 (AUC, 0.85), followed by CAN2 (0.78) and CAN1 (0.70). Age, diabetes duration, and renal function markers were recurrent non-CART predictors, while anthropometric, hematological, metabolic, and treatment-related features showed category- and model-specific contributions. Uniform Manifold Approximation and Projection demonstrated substantial overlap among CAN categories, particularly for CAN1.

**Conclusions:**

CARTs remained essential for accurate CAN classification and staging. Routinely available clinical variables provided complementary phenotypic information but were insufficient to robustly distinguish CAN categories without CART data, particularly CAN0 and CAN1. ML may support multidimensional phenotyping and hypothesis generation but cannot replace standardized autonomic testing.

## Introduction

Cardiac autonomic neuropathy (CAN) is a serious complication of type 2 diabetes (T2DM) associated with increased cardiovascular morbidity and mortality (1, 2). The pathogenesis of CAN is multifactorial, involving complex interactions among chronic metabolic disturbances, oxidative stress, vascular and neural dysfunction, which may complicate the identification of individuals at risk (2, 3). This condition is characterized by impaired autonomic regulation of the cardiovascular system and may remain clinically silent in its early stages, despite progressive autonomic dysfunction (3, 4). Cardiovascular autonomic reflex tests (CARTs) are considered the reference methods for the diagnosis and staging of CAN (5). Machine learning (ML) approaches enable the integration and analysis of multidimensional clinical, laboratory, and physiological data, supporting the identification of determinants associated with the onset and progression of CAN (6). The available evidence suggests that ML methods have considerable potential for the early detection of cardiac autonomic neuropathy (7), prediction of CAN occurrence (8), and identification of associated risk factors (9). The integrated analysis of clinical, laboratory, and physiological data may facilitate the development of advanced decision-support systems, ultimately improving personalized risk stratification and management of patients with T2DM. Several established risk factors of CAN have been described, including advancing age and prolonged diabetes duration, elevated glycated hemoglobin (HbA1c) levels, hypertension, obesity, smoking, dyslipidemia, and concomitant diabetic microvascular complications, particularly retinopathy, nephropathy, and peripheral neuropathy (3). However, individuals with T2DM who remain free of CAN despite prolonged disease duration have received considerably less attention. Therefore, the present study aimed to apply ML approaches to a prospectively established registry and retrospectively collected laboratory, demographic, biometric, and clinical history data in order to identify the factors most strongly associated with and predictive of CAN development in patients with T2DM.

## Patients and methods

### QT Registry

This study was conducted using data from the QT Registry (10), a prospective observational database that collects clinical and cardiovascular autonomic function data primarily from patients with diabetes. The registry also enrolls individuals undergoing autonomic assessment for other medical conditions, as well as healthy volunteers. All consecutive eligible patients attending the Endocrinology and Diabetology Outpatient Center of the Department of Medicine, Albert Szent-Györgyi Medical School, University of Szeged, Szeged, Hungary, for routine diabetes screening, evaluation of suspected diabetic neuropathy, or autonomic assessment related to other medical conditions, were invited to participate in the registry. Data were collected continuously and prospectively by trained assistants in the Neuropathy Laboratory. CARTs and anthropometric measurements were performed during the registry visits. Laboratory values were obtained as part of routine clinical care rather than specifically for the registry, and the dates of the laboratory assessments were recorded. All data were prospectively entered into the registry using a standardized case report form. The case report form used for data collection is available online (11).

### Study participants

For the present study, patients with T2DM who were registered in the QT Registry between September 2, 2019 and April 8, 2026, were included in the analysis. As controls, individuals from the same registry during the same period without diabetes and without any abnormal CART results were included; borderline CART results were permitted. All participants who met the eligibility criteria were included, and no otherwise eligible participant was excluded. If multiple assessments were available for the same participant, only the first assessment was included to avoid potential bias related to repeated measurements. Thus, each participant contributed only once to the analysis. For the present analysis, only cardiovascular autonomic reflex test results and relevant clinical history, anthropometric, and laboratory variables were evaluated. A complete list of variables (n=114) and the extent of missing data for variables included in the machine learning analyses is provided in the **Supplementary Material** as **Supplementary Table 1**. Inclusion criteria for the present analysis were the availability of CART data and a confirmed diagnosis of T2DM (12). Control-group eligibility criteria are described above. Data were extracted from the QT Registry, and data analysis for the present study commenced on April 8, 2026.

### Ewing’s five standard cardiovascular reflex tests, autonomic score

CAN was assessed using the five standardized Ewing CARTs (5) performed with the Cardiosys-A01 system (MDE Heidelberg GmbH, Heidelberg, Germany). Reflex tests were performed by measuring the blood pressure and obtaining continuous 6-lead electrocardiographic (ECG) signals. The signals were digitized with a multichannel data acquisition system; the sampling rate was 2 kHz, and the data were stored for later analysis. The Ewing tests are the gold standard for diagnosing autonomic dysfunction; they provide non-invasive, clinically relevant, standardized and reproducible measures of autonomic functions. Three of these tests record heart rate changes when performing specific activities, while the remaining two measure changes in blood pressure. Heart rate tests reflect changes in parasympathetic function, while those based on blood pressure responses primarily characterize sympathetic function disturbances (13). Heart rate changes were measured during deep inhalation and exhalation, in lying and standing positions with 30/15 ratio, and during and following a Valsalva maneuver (3). Systolic blood pressure changes were measured after standing up from the supine position, while diastolic changes were recorded during sustained handgrip for 3 minutes. CARTs were scored separately: 0 (normal), 1 (borderline), 2 (abnormal). The overall autonomic score was calculated from the sum of each test result to characterize the severity of autonomic dysfunction. The detailed description of the individual Ewing tests is provided in the **Supplementary Material**.

The diagnosis and staging of CAN were based on the Toronto Consensus criteria (3). Participants with normal CART results were classified as the noCAN group. Subjects without abnormal CARTs but with a borderline result (score 1) in at least one test were categorized as borderline CAN (bCAN). Early CAN (eCAN) was defined as one abnormal CART, definite CAN (dCAN) as at least two abnormal CARTs, and severe CAN (sCAN) as definite CAN accompanied by orthostatic hypotension.

For machine learning analyses, additional categories were created: noCAN and bCAN subjects were combined into the CAN0 group, eCAN subjects constituted the CAN1 group, while dCAN and sCAN subjects were merged into the CAN2 group. To improve the predictive performance of the models, a control group was also included from the registry, consisting of non-diabetic individuals without abnormal CART results (i.e., no autonomic reflex test was scored as abnormal [score 2], although borderline results [score 1] were permitted). The reference ranges and interpretation of the CART results are presented in **Supplementary Table 2**.

### Statistical analysis

An overview of the study design, dataset composition, and machine learning workflow is presented in **Figure 1**. Microsoft Excel was applied for general data handling. Jupyter Notebook (version 6.3.0) was utilized for exploratory data analyses and data processing. Statistical evaluation was performed using Python 3.8.8 and the following packages: NumPy (1.22.4), Pandas (2.0.3), SciPy (1.6.2), XGBoost (2.1.3), SHAP (0.44.1), Optuna (4.5.0) and scikit-learn (1.3.2). Categorical data were analyzed using the Chi-square test, while normally distributed data were assessed with Welch’s t-test or Welch’s ANOVA for multiple categories. Non-normally distributed data were evaluated using the Mann-Whitney U-test or the Kruskal-Wallis test for multiple categories to determine significance. The level of statistical significance was set at p < 0.05. Graphical representations were generated using the Matplotlib (3.7.5) and Seaborn (0.13.2) libraries. Uniform Manifold Approximation and Projection (UMAP) (0.5.2) was used for dimension reduction and two-dimensional visualization of the data (14). UMAP visualization uses two derived dimensions: UMAP1 as the x-axis and UMAP2 as the y-axis. Data for UMAP visualization was z-score normalized and missing data was median imputed. These dimensions are determined by nonlinear transformations; therefore, they do not represent single features of the original data. The graph represents relative distances, where patients with more similar features are clustered close together, whereas patients with more dissimilar features have greater distances between them.

**Figure 1:**
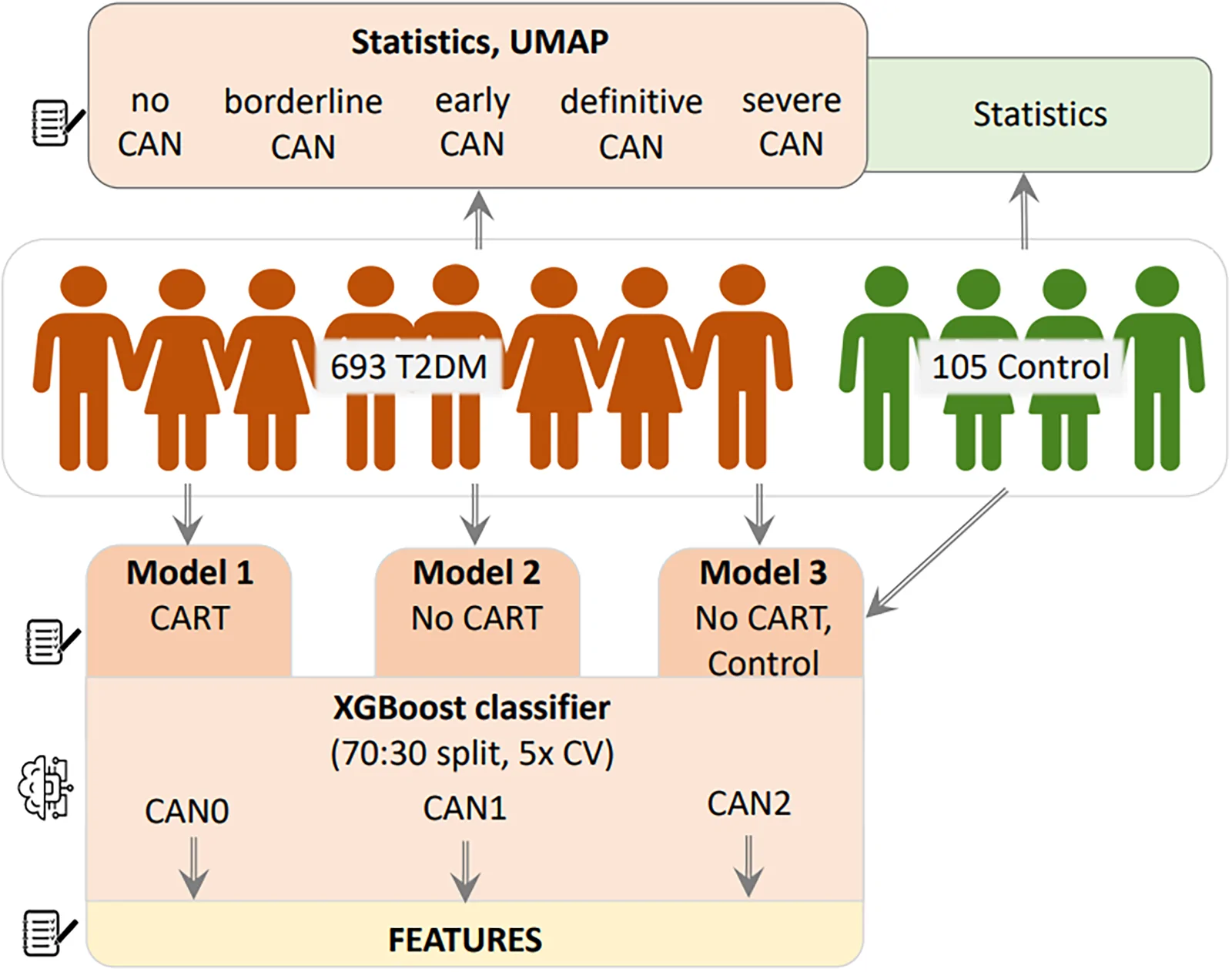
Overview of the study design and machine learning workflow. A total of 693 patients with T2DM were categorized into noCAN, borderline CAN, early CAN, definite CAN, and severe CAN groups. Uniform Manifold Approximation and Projection (UMAP) was used to assess data distribution and homogeneity. Three XGBoost classifiers were developed using different input datasets: (1) including CART features, (2) excluding CART features, and (3) excluding CART features while supplementing the dataset with 105 non-diabetic control participants without abnormal CART results. All models were trained using a 70:30 train–test split and five-fold cross-validation to classify patients into CAN0, CAN1, and CAN2. CAN, cardiac autonomic neuropathy; CAN0, noCAN and borderline CAN combined; CAN1, early CAN; CAN2, definite and severe cardiac autonomic neuropathy combined.

To investigate the features of CAN categories, the supervised tree-based machine learning approach, extreme gradient boosting (XGBoost) was utilized (15). XGBoost was selected for its ability to model nonlinear relationships and interactions, and its native handling of missing values (15). Its compatibility with SHAP-based interpretation further supported its use for exploratory characterization of features associated with CAN categories (16). XGBoost models were built with the dedicated XGBoost library. Key hyperparameters and optimization strategies were predefined. Data were randomly divided into training and testing sets at a 70:30 ratio. Prior to XGBoost classifier model training, class weights were computed and used during training to balance the classes in order to reduce the risk of model bias and overfitting. In every iteration, models were optimized using Optuna [search space: tree depth (2–10), minimum child weight (1–15), learning rate (0.01–0.3), number of trees (100–1500), subsampling ratios (0.5–1.0), and L1/L2 regularization (10^-4^–10)] with 5-fold cross-validation and 100 iterations (17). Missing data were handled natively by XGBoost. Optimization was applied to parameters such as tree depth, child weight, gamma, learning rate, number of estimators, subsample, column sample by tree, level, and node, and regularization terms alpha and lambda. Three XGBoost models were established based on different input data. Model building was iterated through 30 random states (20-49) to collect additional SHAP information on feature stability.

The first model was trained on a dataset including the raw CART data. This model served as a proof of concept; additionally, it ranked the CART features that were important for the determination of CAN categories. The second model omitted CART data, thereby enabling the investigation of other potentially important features. The third model omitted CART data but was supplemented with data from control participants, enabling the investigation of features in a more diverse population. Model interpretability was assessed using SHAP (Shapley Additive Explanations) (0.44.1) values to quantify feature contributions to individual predictions (16). The SHAP method allows a standardized evaluation of ML models, highlighting important features contributing to model performance and providing insight into the “black box” of each model. SHAP results were visualized using beeswarm plots, where the features are displayed on the y-axis, the x-axis represents the contribution of individual feature values to the model, and the color indicates the values of data points. The greater spread along the x-axis generally represents a higher contribution to the model. Color transitions reflect a more linear relationship, whereas mixed colors indicate more complex, non-linear relationships.

## Results

During the study period, a total of 693 CAN assessments performed in patients with T2DM were included in the analysis. Among these, 19 patients (2.7%) exhibited no evidence of autonomic dysfunction, corresponding to an autonomic score of 0. The remaining 674 patients (97.3%) demonstrated at least one borderline or abnormal test result and were assigned an autonomic score ranging from 1 to 10. The distribution of autonomic scores in patients with T2DM is shown in **Figure 2**.

**Figure 2:**
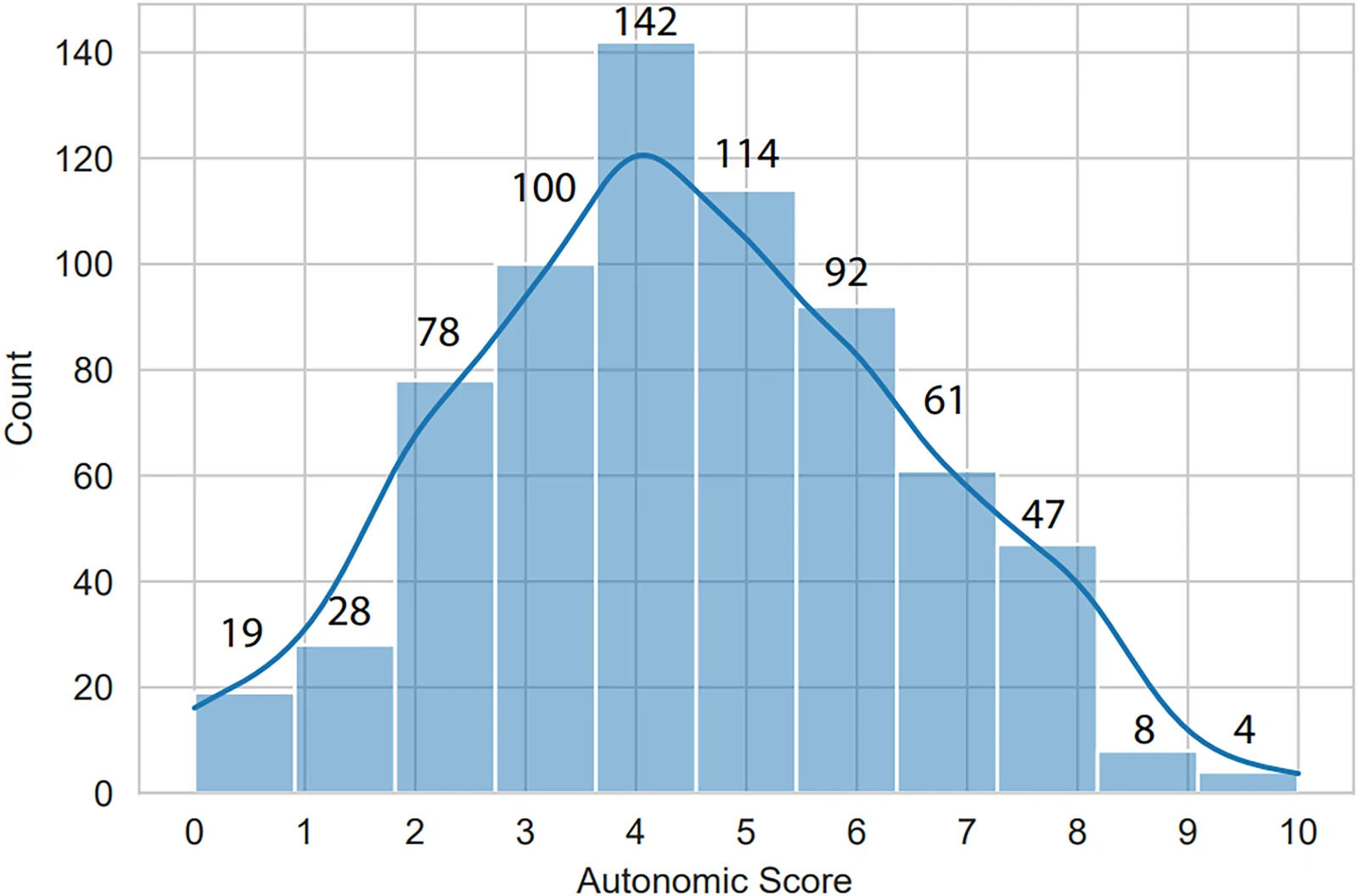
Histogram with kernel density estimation illustrating the distribution of patients (n=693) with T2DM across autonomic score values ranging from 0 to 10. n, number; T2DM, type 2 diabetes.

The cohort included 19 patients with noCAN, 71 with bCAN, 215 with eCAN, 326 with dCAN, and 62 with sCAN, which corresponded to CAN0 (n=90), CAN1 (n=215), and CAN2 (n=388) in the subsequent analyses. Clinical characteristics of patients with T2DM categorized by CAN categories are presented in **Table 1**. Progressive CAN severity was associated with older age, longer diabetes duration, higher systolic blood pressure (all p<0.01), while the prevalence of current smoking differed significantly across CAN categories (p=0.005) without a consistent gradient. In parallel, markers of renal function deteriorated across CAN stages, as reflected by increasing urea and creatinine levels and decreasing estimated glomerular filtration rate (eGFR; all p<0.001). Patients with more advanced CAN also exhibited lower red blood cell count and platelet count (p<0.05), whereas hemoglobin, hematocrit, and albumin did not differ significantly between groups. In contrast, sex distribution, body mass index, glycemic control (HbA1c), and most components of the lipid profile did not differ significantly between groups. Although triglyceride levels differed significantly across CAN stages (p=0.015), the differences did not follow a consistent gradient according to CAN severity. As expected, all cardiovascular autonomic reflex test parameters and the composite autonomic score differed significantly across CAN categories (all p<0.001).

**Table 1:** Clinical characteristics of patients with T2DM categorized by CAN categories.

Characteristics	CAN0		CAN1	CAN2		P-value
	noCAN (n=19)	bCAN (n=71)	eCAN (n=215)	dCAN (n=326)	sCAN (n=62)	
Demographic data						
Male sex	12 (63.2%) (n=19)	32 (45.1%) (n=71)	106 (49.3%) (n=215)	156 (47.9%) (n=326)	37 (59.7%) (n=62)	0.298
Age (years)	51.0 (43.0-64.5) (n=19)	59.0 (51.0-68.0) (n=71)	59.0 (51.0-68.0) (n=215)	66.0 (57.0-73.0) (n=326)	68.0 (59.3-73.8) (n=62)	*<0.001*
Anthropometric data, medical history						
Body mass index (kg/m^2^)	31.4 (26.8-33.1) (n=19)	29.4 (26.3-35.4) (n=71)	32.0 (27.4-36.8) (n=215)	31.2 (27.8-37.0) (n=325)	29.0 (25.8-33.2) (n=62)	0.056
Waist-to-hip ratio	0.96 ± 0.11 (n=15)	0.94 ± 0.11 (n=50)	0.97 ± 0.09 (n=168)	0.96 ± 0.10 (n=231)	1.00 ± 0.09 (n=47)	*0.048*
Systolic blood pressure (mmHg)	126.2 ± 19.46 (n=19)	137.1 ± 16.21 (n=71)	137.9 ± 17.38 (n=214)	139.3 ± 20.16 (n=322)	146.5 ± 15.04 (n=62)	*<0.001*
Diabetes duration (years)	10.4 (5.0-17.0) (n=17)	8.0 (3.5-16.0) (n=65)	10.5 (4.5-19.1) (n=181)	13.6 (6.2-20.7) (n=278)	14.0 (6.8-20.1) (n=52)	*0.008*
Current smoking	4 (21.1%) (n=19)	18 (25.4%) (n=71)	48 (22.5%) (n=213)	38 (11.9%) (n=320)	8 (12.9%) (n=62)	*0.005*
Diet treatment	15 (100.0%) (n=15)	51 (98.1%) (n=52)	162 (97.0%) (n=167)	225 (97.0%) (n=232)	54 (100.0%) (n=54)	0.688
Laboratory parameters						
White blood cell count (G/L)	7.3 (6.4-8.5) (n=13)	7.4 (6.4-9.3) (n=49)	7.4 (6.1-9.3) (n=158)	8.2 (6.8-9.8) (n=226)	7.2 (6.4-9.2) (n=52)	0.050
Red blood cell count (T/L)	4.6 ± 0.48 (n=13)	4.8 ± 0.46 (n=49)	4.8 ± 0.57 (n=158)	4.7 ± 0.58 (n=227)	4.5 ± 0.62 (n=52)	*0.025*
Hemoglobin (g/L)	140.0 (127.0-151.0) (n=13)	143.0 (134.0-148.5) (n=47)	141.0 (130.0-153.5) (n=159)	139.0 (126.5-152.0) (n=227)	135.0 (121.0-147.0) (n=53)	0.125
Hematocrit (%)	40.5 (38.0-43.5) (n=12)	42.0 (39.0-44.0) (n=41)	42.0 (39.0-45.0) (n=135)	41.0 (38.0-45.0) (n=201)	40.0 (37.0-43.0) (n=46)	0.062
MCV (fL)	89.2 (86.2-91.9) (n=13)	87.6 (85.4-90.1) (n=49)	87.0 (83.9-89.9) (n=159)	87.9 (84.1-90.8) (n=225)	88.5 (86.1-91.5) (n=53)	*0.047*
Platelet count (G/L)	244.0 (193.0-304.0) (n=13)	260.0 (212.0-290.3) (n=48)	258.0 (213.0-304.5) (n=159)	256.5 (220.8-294.3) (n=220)	216.0 (188.0-252.5) (n=51)	*0.005*
Urea nitrogen (mmol/L)	4.7 (4.0-6.3) (n=13)	5.6 (4.5-6.7) (n=54)	5.6 (4.8-6.9) (n=153)	6.6 (5.2-8.3) (n=227)	6.3 (5.2-8.1) (n=52)	*<0.001*
Creatinine (µmol/L)	75.0 (56.0-82.0) (n=13)	72.0 (58.0-81.0) (n=53)	75.5 (64.0-91.8) (n=154)	81.0 (69.0-99.0) (n=229)	88.5 (77.5-106.0) (n=52)	*<0.001*
eGFR (mL/min/1.73 m^2^)	102.9 (90.1-118.3) (n=13)	95.1 (75.5-104.7) (n=53)	91.8 (70.1-103.8) (n=154)	77.2 (61.6-96.5) (n=229)	73.6 (57.8-89.8) (n=52)	*<0.001*
Albumin (g/L)	46.5 (41.3-48.0) (n=10)	47.0 (44.8-49.0) (n=36)	46.0 (44.0-49.0) (n=125)	46.0 (43.0-48.0) (n=191)	45.5 (43.0-48.0) (n=36)	0.311
Total cholesterol (mmol/L)	4.9 (4.4-5.7) (n=10)	4.5 (3.6-5.3) (n=40)	4.9 (3.9-5.5) (n=112)	4.6 (3.9-5.4) (n=155)	4.5 (3.4-5.2) (n=36)	0.350
Triglycerides (mmol/L)	1.9 (1.8-2.7) (n=10)	1.3 (1.0-1.9) (n=40)	1.9 (1.4-2.8) (n=114)	2.0 (1.4-2.9) (n=158)	1.7 (1.4-2.7) (n=37)	*0.015*
LDL cholesterol (mmol/L)	2.7 (2.2-3.2) (n=10)	2.1 (1.5-3.2) (n=35)	2.6 (1.7-3.3) (n=93)	2.4 (1.7-3.0) (n=141)	2.1 (1.6-3.4) (n=33)	0.426
HDL cholesterol (mmol/L)	1.2 (0.9-1.7) (n=10)	1.3 (1.0-1.7) (n=36)	1.2 (1.0-1.5) (n=106)	1.2 (1.0-1.5) (n=150)	1.2 (1.0-1.3) (n=34)	0.869
HbA1c (%)	9.4 (8.0-11.1) (n=10)	7.2 (6.6-8.3) (n=37)	7.5 (6.4-8.6) (n=114)	7.5 (6.5-8.8) (n=161)	7.3 (6.0-9.3) (n=33)	0.158
Glucose (mmol/L)	7.8 (5.8-9.1) (n=15)	8.3 (6.5-9.3) (n=52)	7.9 (6.2-10.2) (n=163)	8.1 (6.6-10.5) (n=232)	7.6 (6.0-9.6) (n=52)	0.826
TSH (mIU/L)	2.0 (1.6-2.6) (n=9)	1.7 (1.2-2.6) (n=28)	1.9 (1.2-2.6) (n=97)	1.7 (0.9-2.6) (n=152)	1.6 (1.0-2.4) (n=31)	0.312
C-reactive protein (mg/L)	2.4 (2.1-3.3) (n=8)	2.6 (2.0-9.0) (n=16)	3.6 (2.0-7.0) (n=87)	4.1 (2.0-9.5) (n=141)	3.0 (2.0-8.9) (n=26)	0.630
Medication						
ACEI	5 (26.3%)	10 (14.1%)	32 (14.9%)	66 (20.2%)	9 (14.5%)	0.323
ARB	1 (5.3%)	5 (7.0%)	14 (6.5%)	22 (6.7%)	9 (14.5%)	0.268
Alpha-blockers	N. A.	4 (5.6%)	17 (7.9%)	30 (9.2%)	14 (22.6%)	*0.003*
Beta-blockers	5 (26.3%)	24 (33.8%)	76 (35.3%)	146 (44.8%)	33 (53.2%)	*0.019*
Centrally acting antihypertensives	N. A.	4 (5.6%)	19 (8.8%)	46 (14.1%)	14 (22.6%)	*0.004*
Diuretics	3 (15.8%)	7 (9.9%)	30 (14.0%)	52 (16.0%)	16 (25.8%)	0.128
Insulins	9 (47.4%)	8 (11.3%)	41 (19.1%)	59 (18.1%)	14 (22.6%)	*0.009*
Metformin	12 (63.2%)	42 (59.2%)	102 (47.4%)	156 (47.9%)	32 (51.6%)	0.299
SGLT2 inhibitors	5 (26.3%)	13 (18.3%)	37 (17.2%)	61 (18.7%)	11 (17.7%)	0.904
DPP-4 inhibitors	N. A.	2 (2.8%)	1 (0.5%)	16 (4.9%)	2 (3.2%)	0.053
GLP-1 receptor agonists	2 (10.5%)	12 (16.9%)	40 (18.6%)	58 (17.8%)	18 (29.0%)	0.239
Statins	6 (31.6%)	26 (36.6%)	80 (37.2%)	143 (43.9%)	30 (48.4%)	0.284
Antiplatelet drugs	1 (5.3%)	14 (19.7%)	49 (22.8%)	112 (34.4%)	27 (43.5%)	*<0.001*
Alpha-lipoic acid	5 (26.3%)	11 (15.5%)	44 (20.5%)	107 (32.8%)	25 (40.3%)	*<0.001*
Neuropathy medications	N. A.	3 (4.2%)	13 (6.0%)	35 (10.7%)	4 (6.5%)	0.103
CARTs						
HR response to deep breathing (beats/min)	36.0 (25.5-44.5) (n=19)	17.0 (14.0-27.5) (n=71)	16.0 (11.0-21.0) (n=215)	8.0 (5.0-14.0) (n=325)	9.0 (6.3-14.0) (n=62)	*<0.001*
Valsalva ratio	1.6 (1.4-1.8) (n=19)	1.3 (1.2-1.5) (n=71)	1.3 (1.2-1.4) (n=214)	1.2 (1.1-1.3) (n=320)	1.2 (1.1-1.3) (n=62)	*<0.001*
30:15 ratio	1.2 (1.1-1.2) (n=19)	1.1 (1.0-1.1) (n=68)	1.0 (1.0-1.1) (n=208)	1.00 (0.98-1.0) (n=315)	1.00 (0.96-1.0) (n=61)	*<0.001*
SBP response to standing (mmHg)	2.0 (0.0-6.0) (n=19)	5.0 (0.0-15.0) (n=69)	3.0 (0.0-11.0) (n=208)	2.0 (0.0-9.0) (n=313)	29.0 (24.0-37.0) (n=62)	*<0.001*
DBP response during sustained handgrip (mmHg)	21.0 (19.5-24.0) (n=19)	15.0 (12.5-20.5) (n=71)	12.0 (6.0-19.0) (n=182)	7.0 (3.0-11.8) (n=222)	11.5 (5.0-19.0) (n=50)	*<0.001*
Autonomic score	0.0 (0.0-0.0) (n=19)	2.0 (1.0-2.0) (n=71)	3.0 (2.0-4.0) (n=215)	5.0 (4.0-6.0) (n=326)	6.0 (5.0-7.0) (n=62)	*<0.001*

ACEI, angiotensin-converting enzyme inhibitors; ARB, angiotensin II receptor blockers; bCAN, borderline cardiac autonomic neuropathy; CAN, cardiac autonomic neuropathy; CAN0, noCAN and bCAN combined; CAN1, early CAN; CAN2, definite and severe cardiac autonomic neuropathy combined; CART, cardiovascular autonomic reflex test; DBP, diastolic blood pressure; dCAN, definite cardiac autonomic neuropathy; DPP-4 inhibitors, dipeptidyl peptidase-4 inhibitors; eCAN, early cardiac autonomic neuropathy; eGFR, estimated glomerular filtration rate; GLP-1 receptor agonists, glucagon-like peptide-1 receptor agonists; HR, heart rate; HbA1c, glycated hemoglobin; HDL, high-density lipoprotein cholesterol; LDL, low-density lipoprotein cholesterol; MCV, mean corpuscular volume; N.A., no data available; noCAN, no cardiac autonomic neuropathy; sCAN, severe cardiac autonomic neuropathy; SBP, systolic blood pressure; SGLT2 inhibitors, sodium-glucose co-transporter 2 inhibitors; TSH, thyroid-stimulating hormone; T2DM, type 2 diabetes mellitus. Categorical variables are presented as n (%). Variables with a normal distribution are presented as mean ± standard deviation (SD), whereas non-normally distributed variables are reported as median (interquartile range [IQR]). Italicized p-values indicate statistically significant results.

Medication use varied significantly across CAN severity groups. The prevalence of beta-blocker therapy increased progressively with CAN severity (p=0.019). In contrast, no significant differences were observed for angiotensin II receptor blockers (ARBs) or diuretics, whereas the use of centrally acting antihypertensive agents (p=0.004) and alpha-blockers (p=0.003) was significantly more frequent in patients with more advanced CAN. In addition, the use of alpha-lipoic acid (p<0.001) and antiplatelet drugs (p<0.001) also increased with CAN severity. No significant differences were observed for neuropathy medications. No significant differences were observed for ACE inhibitors, sodium–glucose cotransporter 2 (SGLT2) inhibitors, dipeptidyl peptidase-4 (DPP-4) inhibitors, glucagon-like peptide-1 (GLP-1) receptor agonists, or statins.

The relatively high number of patients and the multidimensionality allowed us to investigate the structure and homogeneity of the data; therefore, UMAP was used for dimensionality reduction and two-dimensional visualization (**Figure 3**). Overall, the dataset was quite homogeneous, as the projection produced a single large cluster. Mapping the CAN categories to the UMAP projection, partial enrichment of specific regions rather than distinct cluster separation was observed. NoCAN and bCAN map to the top left region, whereas dCAN and sCAN are located in the lower right area. In contrast, eCAN was broadly distributed across the projection, substantially overlapping with the other CAN categories.

**Figure 3:**
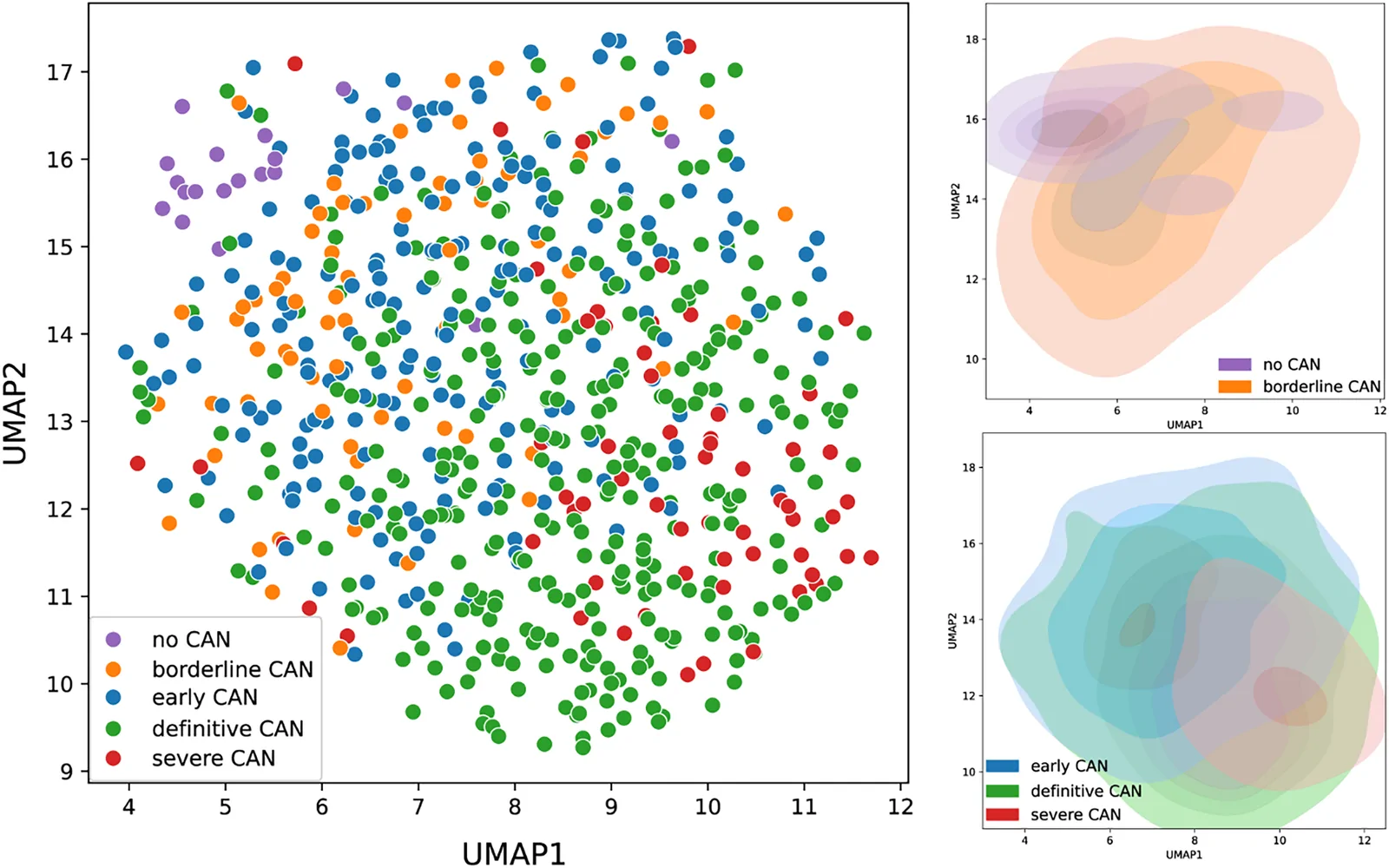
Uniform manifold approximation and projection (UMAP) projection of the T2DM dataset, with colors indicating cardiovascular autonomic neuropathy (CAN) categories. Smaller plots show kernel density estimations for each CAN category. T2DM, type 2 diabetes.

To further investigate the features contributing to CAN classification in this high-dimensional dataset, we decided to further analyze the data with the decision tree-based method XGBoost, for which data dimensionality and collinearity have only a moderate influence on performance. The list of registry-derived features used for model development is provided as **Supplementary Material** in **Supplementary Table 1**. Models were built using three datasets, and all models were trained to classify patients into 3 different CAN categories (CAN0, CAN1, and CAN2). All models were run using 30 different random states to obtain a better overview of the SHAP values (**Supplementary Table 3**). In the first model, we validated the approach by providing the five measured variables required for autonomic score calculation (CART data) as input features alongside the other registry-derived features. The training data did not include the reference ranges used for interpretation of the test results (**Supplementary Table 2**). This model serves as a test for XGBoost performance, using CART data the model should be able to achieve high performance because of the relatively simple mathematical relations between CART and CAN outputs. As expected, the model performed exceptionally well (**Figure 4**; **Supplementary Figure 1**, **2**, **Supplementary Table 3**, **4**). The leading SHAP-based feature profiles for the CAN0 and CAN2 categories were similar (CART data, age, serum creatinine, waist-to-hip ratio,…). Interestingly, the SHAP values for the CAN1 category were an order of magnitude lower; therefore, CART data were less dominant. The best CART-based multiclass XGBoost model achieved excellent discrimination between CAN categories, with an overall accuracy of 100% and a weighted AUC of 1.00. All five CARTs ranked among the leading predictors in each CAN category, although their relative importance varied. In CAN0, former smoking duration, defined as the cumulative years of smoking among former smokers, ranked unexpectedly high, followed by age and renal, anthropometric, inflammatory, and metabolic variables. The non-CART profile shifted toward hematological and metabolic parameters, body mass index (BMI), diabetes duration, and liver enzymes in CAN1, and toward age, renal markers, and hepatic, endocrine, anthropometric, and systolic blood pressure variables in CAN2. No non-CART feature was prominent across all three categories; however, age, serum creatinine, and waist-to-hip ratio recurred in CAN0 and CAN2; triglycerides and mean corpuscular volume in CAN0 and CAN1; and white blood cell count, alkaline phosphatase, and systolic blood pressure in CAN1 and CAN2.

**Figure 4:**
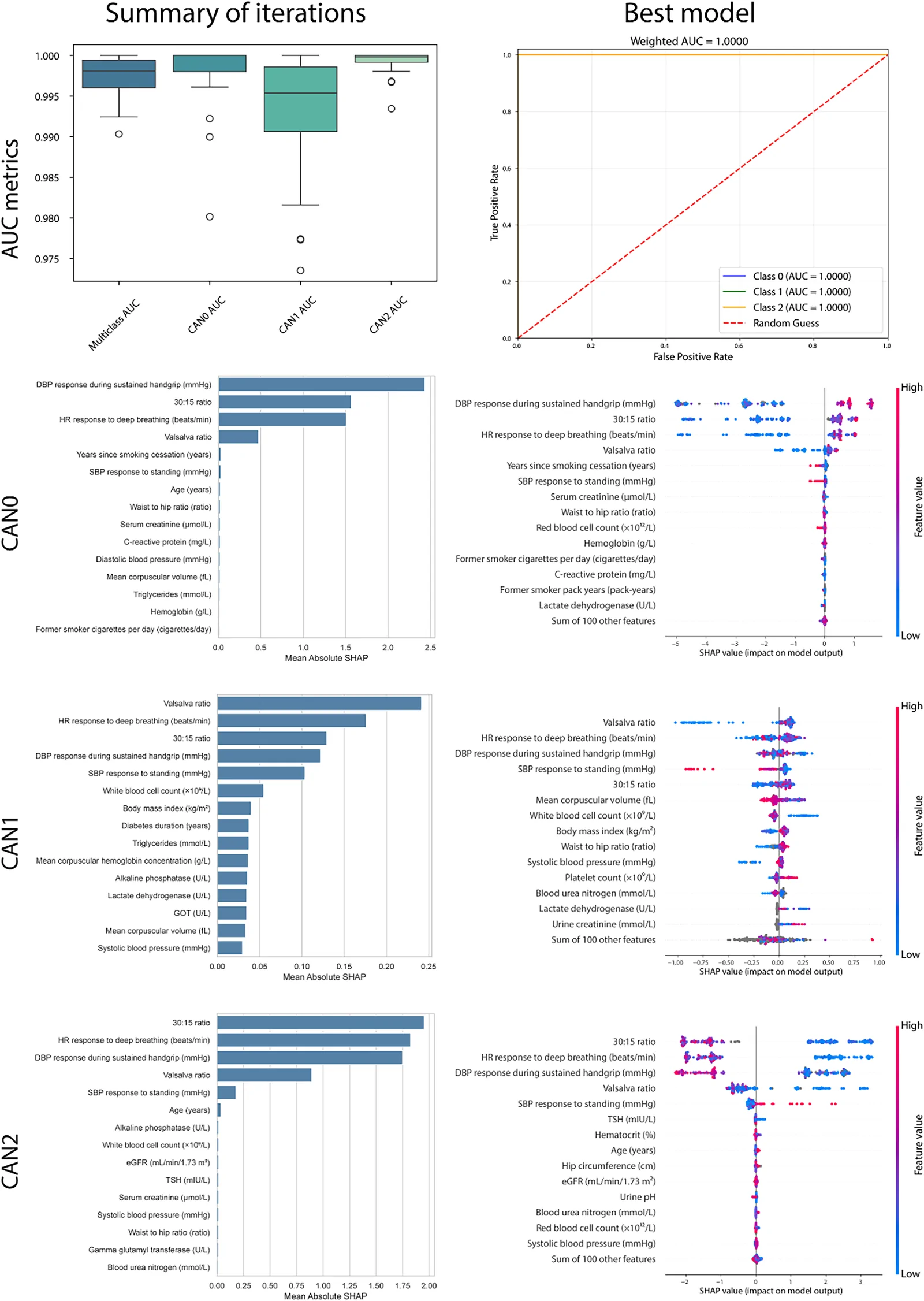
Representation of the first XGBoost model trained on the T2DM dataset, with CART data included. The classification model was trained to predict CAN categories. The first column summarizes the XGBoost results across 30 random states. The box plot indicates the AUC scores, and the bar charts show the 15 highest mean absolute SHAP values corresponding to the CAN0, CAN1, and CAN2 categories. The second column represents the best model. The ROC curves indicate the AUC for each class. Beeswarm plots represent the most important predictors based on SHAP values. Further model statistics are available in **Supplementary Table 3**, **4**.

Next, the data required for autonomic score calculations (CARTs) were omitted to identify other important features. The second classification model was similarly used to distinguish among the CAN0, CAN1, and CAN2 categories. With this approach, the model performance dropped drastically, and the best model reached a weighted AUC of 0.64 (**Figure 5**; **Supplementary Figure 1**, **2**, **Supplementary Table 3**, **4**). In this second model, the class-specific AUC was highest for CAN2 (0.68), whereas CAN0 and CAN1 were predicted only slightly better than random (AUC, 0.59 for both) (**Figure 5**). The second model was further investigated using SHAP analysis to highlight the most relevant features of the data (**Figure 5**). The CAN2 category showed more distinctive predictive features, whereas CAN0 and CAN1 features had lower SHAP values, explaining the poor predictive performance of the model. In CAN0, prediction was driven mainly by metabolic, inflammatory, and renal markers, together with diabetes duration and anthropometric variables. In CAN1, the profile shifted toward hematological, anthropometric, hepatic, and glycemic parameters. In CAN2, age was the dominant predictor, accompanied by blood pressure, thyroid and renal markers, treatment-related variables, and liver disease. Age, diabetes duration, and blood urea nitrogen contributed across all three categories, while other predictors showed category-specific overlap.

**Figure 5:**
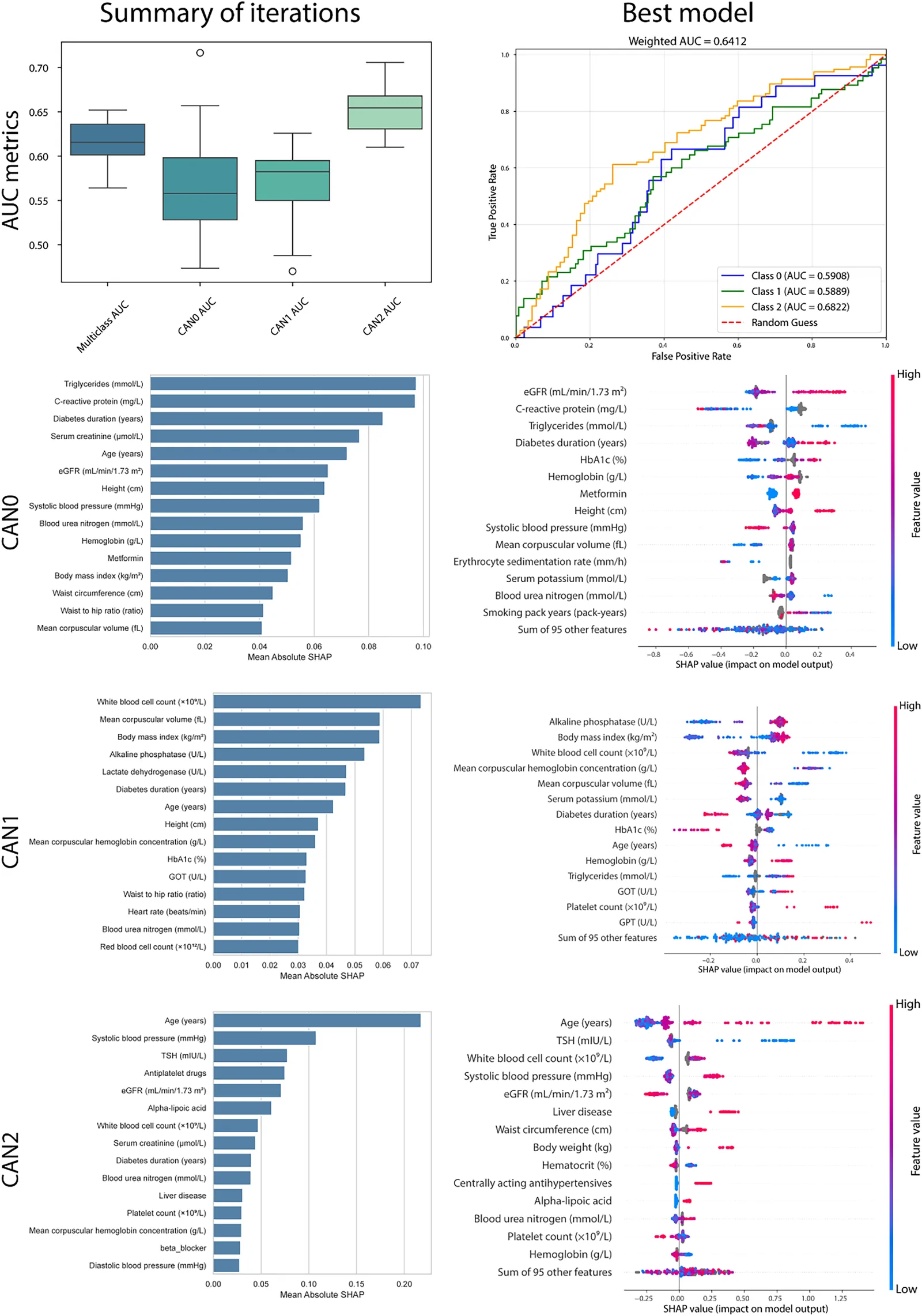
Representation of the second XGBoost model trained on the T2DM dataset, with CART data excluded. The classification model was trained to predict CAN categories. The first column summarizes the XGBoost results across 30 random states. The box plot indicates the AUC scores, and the bar charts show the 15 highest mean absolute SHAP values corresponding to the CAN0, CAN1, and CAN2 categories. The second column represents the best model. The ROC curves indicate the AUC for each class. Beeswarm plots represent the most important predictors based on SHAP values. Further model statistics are available in **Supplementary Table 3**, **4**.

Next, we were interested in whether additional data could improve the classification of the CAN0 and CAN1 categories. The dataset of patients with T2DM was supplemented with a control group with no diabetes and no early, definite, or severe CAN. The comparison of the clinical characteristics between the T2DM population and the included controls is shown in **Supplementary Table 5**. The performance of the best model of the third approach reached a weighted AUC of 0.78 (**Figure 6**; **Supplementary Figure 1**, **2**, **Supplementary Table 3**, **4**). Notably, classification performance improved not only for CAN0 and CAN1 but also for CAN2. Feature importances were similar to those identified previously (e.g., age and diabetes duration). After inclusion of the control group, the best model achieved a weighted AUC of 0.78, with the highest class-specific AUC for CAN0 (0.85), followed by CAN2 (0.78) and CAN1 (0.70). CAN0 prediction was driven primarily by diabetes duration, age, and diet therapy, followed by anthropometric, metabolic, inflammatory, renal, and hemodynamic variables. In CAN1, diabetes duration and anthropometric measures predominated, accompanied by hematological, treatment-related, and hepatic parameters. In CAN2, age was the dominant predictor, followed by systolic blood pressure and diabetes duration, with additional contributions from thyroid, renal, treatment-related, and hematological variables. Diabetes duration, systolic blood pressure, blood urea nitrogen, BMI, and heart rate contributed across all three categories.

**Figure 6:**
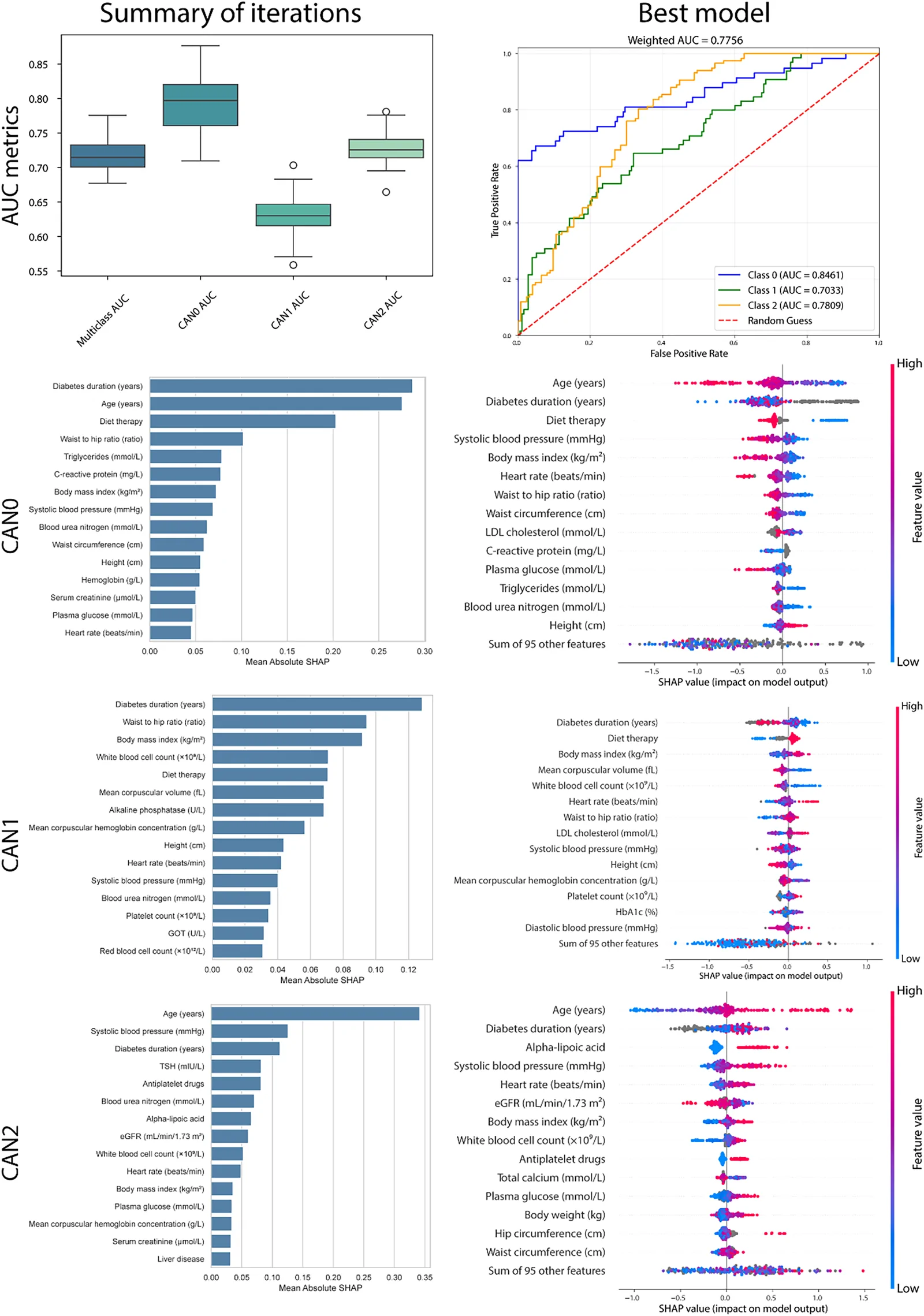
Representation of the third XGBoost model trained on the T2DM dataset, with CART data excluded and the dataset supplemented with control data. The classification model was trained to predict CAN categories. The first column summarizes the XGBoost results across 30 random states. The box plot indicates the AUC scores, and the bar charts show the 15 highest mean absolute SHAP values corresponding to the CAN0, CAN1, and CAN2 categories. The second column represents the best model. The ROC curves indicate the AUC for each class. Beeswarm plots represent the most important predictors based on SHAP values. Further model statistics are available in **Supplementary Table 3**, **4**.

Neither model calibration nor external validity was assessed. An independent dataset with comparable CART measurements, CAN classification, and clinical variables was not available to us. Accordingly, these models should be regarded as exploratory tools for phenotypic characterization; their predicted probabilities and performance cannot be assumed to transfer reliably to other clinical settings. Calibration assessment and external validation are required before clinical application.

## Discussion

In this study, machine learning analysis of a large, clinically characterized T2DM cohort demonstrated that CAN severity was associated with a multidimensional clinical and cardiometabolic profile. As expected, CARTs dominated classification because these tests defined the CAN categories; however, the integration of clinical, laboratory, anthropometric, and treatment-related variables provided additional insights into features associated with different CAN stages.

We analyzed 693 patients with T2DM referred to our neuropathy laboratory and included in the QT registry. In this clinically referred cohort, borderline or abnormal CART findings were highly prevalent: only 2.7% of patients showed completely normal autonomic function, while the vast majority (97.3%) exhibited at least one borderline or abnormal CART result (**Figure 2**). This high proportion includes borderline findings and should not be interpreted as the prevalence of established CAN. Recruitment from a single university-based neuropathy laboratory introduces potential selection and referral bias, as individuals undergoing autonomic assessment may differ systematically from the broader T2DM population. Consequently, the observed frequency of CART abnormalities cannot be extrapolated to unselected patients with T2DM. This selected clinical spectrum may also influence the identified feature associations and model performance, substantially limiting their generalizability to primary care and population-based screening settings. External validation in independent, less selected T2DM cohorts is therefore needed before broader application.

Increasing CAN severity was accompanied by differences in several clinical and biochemical parameters. Older age, longer diabetes duration, and higher systolic blood pressure were observed across increasing CAN categories. In parallel, renal function was poorer in the more advanced CAN categories, as reflected by higher creatinine and blood urea nitrogen levels and lower estimated glomerular filtration rate (**Table 1**). These findings reinforce the close relationship between CAN and diabetes-related renal impairment, potentially reflecting shared microvascular pathways.

Medication patterns further mirrored disease progression. The use of beta-blockers, centrally acting antihypertensive agents, alpha-blockers, alpha-lipoic acid, and antiplatelet therapies increased significantly with worsening CAN stage (**Table 1**). This likely reflects differences in treatment patterns associated with CAN severity, although the cross-sectional design precludes causal interpretation.

Dimensionality reduction using UMAP revealed a largely homogeneous T2DM population structure; however, partial regional enrichment of CAN categories was observed (**Figure 3**). This suggests that despite overlapping clinical features, CAN categories show limited spatial patterning in latent feature space.

This study extends previous machine learning analyses of CAN in patients with T2DM incorporating comprehensive anthropometric, clinical, comorbidity, laboratory, and medication data. The first CART-based XGBoost models (**Figure 4**) demonstrated near-perfect discrimination between CAN severity categories, with the best model achieving weighted AUC of 1.00 and an overall accuracy of 100%. Because the CAN categories were derived from the same CART measurements included as predictors, this analysis involves inherent circularity. The perfect discrimination achieved in the best iteration therefore reflects reconstruction of the diagnostic classification rather than independent predictive validity. The CART-inclusive model should be regarded as a descriptive reference, not as evidence of clinical utility or generalizability. The heart rate response to deep breathing, Valsalva ratio, and 30/15 ratio were consistently influential, while the sustained handgrip response also ranked prominently, particularly in CAN0 and CAN2; the systolic blood pressure response to standing generally contributed less. The higher importance of heart rate-based reflex tests in our model is consistent with the findings of Ewing et al., who demonstrated that heart rate abnormalities occur earlier and more frequently than blood pressure abnormalities during CAN progression (13). The prominent contribution of the sustained handgrip response, despite its known dependence on patient compliance and weaker concordance with other established autonomic function tests, warrants cautious interpretation (18, 19).

When CART variables were excluded, model performance markedly declined (best weighted AUC, 0.64), as expected given that the CAN categories were defined by CART results (**Figure 5**). In this setting, the remaining registry-derived features were insufficient to robustly separate CAN categories: both CAN0 and CAN1 were predicted only slightly better than random, whereas CAN2 showed modestly better discrimination. The combined CAN0 category includes both normal and borderline CART findings, potentially obscuring differences between these subgroups. As no sensitivity analysis excluding borderline cases was performed, the robustness of the findings to this grouping remains untested. The limited discrimination of CAN1 highlights the difficulty of identifying early CAN using routine clinical variables alone. Although the substantial overlap observed on UMAP is consistent with this finding, it does not establish the validity of the grouping. These models should therefore be regarded as exploratory tools for phenotypic characterization rather than validated screening tools for early CAN.

After incorporating a non-diabetic control group, model performance improved (best weighted AUC, 0.78), with the most notable gain observed in the classification of CAN0 (**Figure 6**). This finding suggests that the inclusion of individuals without diabetes enhanced discrimination of the CAN0 category. However, it is important to note that the inclusion of the control group extended the study population beyond patients with T2DM; therefore, the performance of this model is not directly comparable with that of the T2DM-only model.

The prominent contribution of age and diabetes duration was anticipated, as both are well-established risk factors for the development and progression of CAN in patients with T2DM (3, 20, 21), consistent with the findings of Nedergaard et al. (6). At least one of these two variables was identified in every model across all CAN categories.

Anthropometric variables, particularly BMI and waist-to-hip ratio, appeared among the leading predictors in at least one CAN category in each of the three models. Their association with CAN is well documented in the literature (22–24).

Treatment-related predictors were most evident in the CART-excluded models. In the T2DM-only model, metformin contributed to CAN0 classification, although its isolated occurrence in this model warrants cautious interpretation, whereas alpha-lipoic acid, antiplatelet therapy, beta-blocker use, and centrally acting antihypertensive treatment contributed to CAN2 prediction. The observed association between centrally acting antihypertensive agents (rilmenidine, moxonidine, and methyldopa) and CAN is likely confounded by indication, as these drugs are typically used as later-line add-on therapies in patients with more complex or resistant hypertension, which may coexist with more advanced autonomic dysfunction. Moreover, hypotension is a recognized adverse effect of all three agents (25), while being a less common side effect of moxonidine and rilmenidine (26), and as a defining feature of severe CAN, their use may further confound or amplify the clinical assessment of autonomic dysfunction severity. After inclusion of non-diabetic controls, alpha-lipoic acid and antiplatelet therapy remained relevant to CAN2, while diet therapy emerged as an important predictor of CAN0 and CAN1. These associations likely reflect underlying treatment patterns and confounding by indication rather than independent medication effects: neuropathy-directed and cardiovascular therapies may indicate more advanced neurological or vascular disease, whereas diet therapy may distinguish patients with T2DM from non-diabetic controls. This finding is also consistent with current Hungarian diabetes management guidelines, which recommend medical nutrition therapy as a fundamental component of treatment for all patients with diabetes, irrespective of pharmacological therapy (27). As diet therapy was substantially less frequent among control participants than among patients with T2DM, the model may have used this variable primarily as a marker of diabetes status. These category- and model-specific contributions therefore warrant cautious interpretation.

Using an ensemble machine learning approach, Nedergaard et al. identified heart rate variability indices, inflammatory biomarkers (e.g., interleukin-2), diabetes duration, and age as the key predictors of CAN in patients with T2DM (6). In our analysis, C-reactive protein consistently contributed to CAN0 classification and was particularly prominent in the CART-excluded T2DM-only model. Conversely, white blood cell count contributed predominantly to CAN1 and CAN2 across the models, ranking as the leading predictor of CAN1 in the CART-excluded T2DM-only model. Erythrocyte sedimentation rate showed only a limited, model-specific contribution. Other hematological features, including red blood cell count, hemoglobin, hematocrit, platelet count, and erythrocyte indices, recurred across CAN categories, although their importance varied between models.

Glycemic markers showed limited and category-specific contributions. HbA1c was not a leading predictor in the CART-inclusive model but contributed primarily to CAN1 classification in the CART-excluded analyses, with an additional model-specific contribution to CAN0. Plasma glucose became relevant mainly after inclusion of non-diabetic controls, contributing to CAN0 and CAN2 classification. Among lipid variables, triglycerides were the most recurrent predictor, contributing to CAN0 across all three models and to CAN1 in selected analyses. Low-density lipoprotein (LDL) cholesterol showed limited contributions to CAN0 and CAN1 after control-group inclusion, whereas high-density lipoprotein (HDL) cholesterol did not rank among the leading features. These findings indicate model- and category-dependent metabolic contributions rather than a uniform association with increasing CAN severity. Although previous evidence has linked poor glycemic control and dyslipidemia, particularly low HDL cholesterol and elevated triglycerides, to CAN (28), the present SHAP results reflect contributions to model predictions and should not be interpreted as evidence of independent or causal effects.

Renal function markers were among the most consistent non-CART predictors. Estimated glomerular filtration rate (eGFR), blood urea nitrogen, and serum creatinine recurred across all three models and CAN categories, although their relative importance varied. This pattern is consistent with the close relationship between CAN and renal impairment in T2DM, potentially reflecting overlapping microvascular pathways, and with previous reports of associations with albuminuria, reduced eGFR, and accelerated renal function decline (29, 30).

Thyroid-stimulating hormone (TSH) emerged as a recurrent predictor of CAN2 across all three models, although its relative importance varied. Notably, TSH levels did not differ significantly across CAN categories in the conventional statistical analysis, suggesting that the machine learning models may have captured nonlinear effects or interactions with other features rather than a simple univariate association. Previous studies have suggested a relationship between thyroid-axis alterations and autonomic nervous system function (31), but evidence specifically linking TSH to diabetic CAN remains limited. Accordingly, its consistent contribution to CAN2 classification should be regarded as hypothesis-generating rather than evidence of an independent or causal association.

## Conclusion

Collectively, our findings support a model of CAN as a multidimensional phenotype embedded within systemic cardiometabolic disease. This aligns with previous evidence indicating that CAN is strongly associated with both microvascular and macrovascular complications in diabetes. The observed association between CAN severity and renal dysfunction is consistent with shared pathophysiological mechanisms involving microvascular injury, oxidative stress, and autonomic dysregulation, although these mechanisms were not directly assessed. Autonomic neuropathy in diabetes has been increasingly recognized as part of a systemic neurovascular complication spectrum rather than an isolated entity.

From a clinical perspective, these findings emphasize that CAN should be regarded as a systemic complication of T2DM rather than an isolated manifestation of autonomic dysfunction. Beyond confirming the central diagnostic value of CARTs, our results indicate that routinely available demographic, clinical, laboratory, and treatment-related variables contain complementary information; however, these variables were insufficient to robustly distinguish CAN categories without CART data, particularly CAN0 and CAN1. Machine learning offers a practical framework for integrating these multidimensional data. From a methodological perspective, XGBoost demonstrated strong performance when CART variables were included, while UMAP visualization revealed substantial overlap of early CAN with other phenotypes, providing a plausible explanation for the lower classification performance observed in this disease stage. CAN is a prevalent complication in this clinically referred T2DM cohort. While CARTs remain the diagnostic cornerstone, machine learning may support multidimensional phenotyping and hypothesis generation but cannot replace standardized autonomic testing for CAN diagnosis and staging.

## Data Availability

The de-identified data supporting the findings of this study are available from the corresponding author upon reasonable request, subject to applicable data protection regulations and ethical restrictions.

## Glossary

ACEI: angiotensin-converting enzyme inhibitor
ANOVA: analysis of variance
ARB: angiotensin II receptor blocker
AUC: area under the receiver operating characteristic curve
bCAN: borderline cardiovascular autonomic neuropathy
BMI: body mass index
CAN: cardiovascular autonomic neuropathy
CAN0: no CAN and borderline CAN combined
CAN1: early CAN
CAN2: definite and severe CAN combined
CART: cardiovascular autonomic reflex test
DBP: diastolic blood pressure
dCAN: definite cardiovascular autonomic neuropathy
DPP-4: dipeptidyl peptidase-4
ECG: electrocardiogram
eCAN: early cardiovascular autonomic neuropathy
eGFR: estimated glomerular filtration rate
GLP-1: glucagon-like peptide-1
GOT: glutamate oxaloacetate transaminase
GPT: glutamate pyruvate transaminase
HbA1c: glycated hemoglobin
HDL: high-density lipoprotein cholesterol
HR: heart rate
IQR: interquartile range
LDL: low-density lipoprotein cholesterol
MCV: mean corpuscular volume
ML: machine learning
noCAN: no cardiovascular autonomic neuropathy
ROC: receiver operating characteristic
SBP: systolic blood pressure
sCAN: severe cardiovascular autonomic neuropathy
SD: standard deviation
SGLT2: sodium–glucose cotransporter 2
SHAP: SHapley Additive exPlanations
T2DM: type 2 diabetes mellitus
TSH: thyroid-stimulating hormone
UMAP: Uniform Manifold Approximation and Projection
XGBoost: extreme gradient boosting.
