# Supplementary material for: Machine learning predictors of cardiovascular autonomic neuropathy in type 2 diabetes: insights from a prospectively collected single-center registry
Source: Front Endocrinol (Lausanne). 2026 Oct 15;17:1965013. doi: 10.3389/fendo.2026.1965013 (PMC13716568; doi:10.3389/fendo.2026.1965013)
Supplement: Supplementary file 1 [file SupplementaryFile1.zip › Supplementary material.DOCX]

**Machine learning predictors of cardiovascular autonomic neuropathy in type 2 diabetes: insights from a prospectively collected single-center registry**

**Katalin Farkas^1^**†**, Viktor Vedelek^2^**†**, Máté Vámos^1^, Éva Csajbók^1^**, **István Baczkó^3^, János Zádori^4^, Andrea Szentesi^5^, Péter Hegyi^5,6,7,8^, Tamás Várkonyi^1^, Csaba Lengyel^1^**††**, Anna Vágvölgyi^1^***††

**SUPPLEMENTARY MATERIAL**

**Assessment of Ewing’s five standard cardiovascular reflex tests (CARTs)** (5)

**Heart rate response to deep breathing**

Heart rate physiologically increases during inspiration and decreases during expiration. Participants performed paced deep breathing at six cycles per minute (5 seconds inhalation and 5 seconds exhalation), and the difference between the maximum and minimum heart rate (beats/min) was calculated across six respiratory cycles.

**Heart rate response to standing up (30/15 ratio)**

Upon transition from the supine to the standing position, heart rate normally rises rapidly, reaching its maximum around the 15^th^ heartbeat. This initial tachycardic response is followed by a transient slowing of the heart rate in healthy individuals, with the lowest rate typically occurring near the 30^th^ beat. During the assessment, participants rested in a supine position before being instructed to stand, while continuous ECG monitoring was performed throughout the maneuver. The 30/15 ratio was determined by dividing the longest R–R interval observed around the 30^th^ heartbeat by the shortest R–R interval recorded near the 15^th^ heartbeat after standing.

**Heart rate response to the Valsalva maneuver (Valsalva-ratio)**

During the Valsalva maneuver, healthy individuals typically exhibit a decrease in blood pressure and an increase in heart rate, followed by a blood pressure overshoot and heart rate reduction after release. Participants exhaled into a manometer and maintained a pressure of 40 mmHg for 15 seconds while continuous electrocardiographic (ECG) recording was performed. The Valsalva ratio was calculated as the longest post-maneuver R–R interval divided by the shortest R–R interval recorded during the maneuver.

**Systolic blood pressure response to positional change**

In healthy individuals, blood pooling in the lower extremities upon standing is rapidly counteracted by peripheral vasoconstriction. Orthostatic hypotension is a characteristic manifestation of cardiovascular autonomic neuropathy. Blood pressure was measured after 10 minutes of supine rest and at 1, 5, and 10 minutes following standing. The orthostatic response was defined as the greatest decrease in systolic blood pressure compared with the supine value.

**Diastolic blood pressure response during sustained handgrip**

The sustained handgrip test evaluates changes in diastolic blood pressure during isometric exercise. Maximum voluntary grip strength was first determined using a hand-held dynamometer, after which participants maintained a contraction at 30% of maximal force for 3 minutes. Blood pressure was measured every minute on the contralateral arm, and the highest increase in diastolic blood pressure was recorded as the test response.

**Supplementary Figure 1. Boxplots represent model metric distribution of iterations.**

**Supplementary Figure 2. Boxplot represent model Multiclass ROC-AUC Score.**

**Supplementary Table 1 Features utilized, and missingness.** (Supplementary Table 1.xlsx)

**Supplementary Table 2.** **Reference ranges for cardiovascular autonomic reflex tests (CARTs)** (5).

| CARTs | Normal (0 point) | Borderline (1 point) | Abnormal (2 points) |
| --- | --- | --- | --- |
| HR response to deep breathing (beats/min) | ≥15 | >10 to <15 | ≤10 |
| Valsalva ratio | ≥1.21 | >1.10 to <1.21 | ≤1.10 |
| 30:15 ratio | ≥1.04 | >1.00 to <1.04 | ≤1.00 |
| SBP response to standing (mmHg) | ≤10 | >10 to <30 | ≥30 |
| DBP response during sustained handgrip (mmHg) | ≥16 | >10 to <16 | ≤10 |

*Abbreviations: CARTs, cardiovascular autonomic reflex tests; DBP, diastolic blood pressure; HR, heart rate; SBP, systolic blood pressure.*

**Supplementary Table 3 Table summarizes SHAP metrics for each model: absolute SHAP values and occurrence of predictors in each CAN class (feature stability).** (Supplementary Table 3.xlsx)

**Supplementary Table 4 Supplementary material contain model metrics of all three models with all their iterations. Metrics included: AUC score, confusion matrix, accuracy, recall, F1 and specificity.** (Supplementary Table 4.xlsx)

**Supplementary Table 5.** **Comparison of clinical characteristics between control participants and patients with type 2 diabetes.**

| CHARACTERISTICS | Controls  (n=105) | Patients with T2DM  (n=693) | P-value |
| --- | --- | --- | --- |
| DEMOGRAPHIC DATA | |  |  |
| Male sex | 36 (34.3%)  (n=105) | 343 (49.5%)  (n=693) | *0.005* |
| Age (years) | 34.85 ± 13.52  (n=105) | 61.97 ± 12.29  (n=693) | *<0.001* |
| ANTHROPOMETRIC DATA | |  |  |
| Body mass index (kg/m^2^) | 25.92 ± 6.11  (n=105) | 32.38 ± 9.19  (n=692) | *<0.001* |
| Weight (kg) | 75.92 ± 18.51  (n=105) | 91.29 ± 22.92  (n=692) | *<0.001* |
| Height (cm) | 171.15 ± 9.58  (n=105) | 168.01 ± 10.62  (n=692) | *0.008* |
| Waist (cm) | 84.97 ± 17.12  (n=100) | 103.71 ± 18.93  (n=511) | *<0.001* |
| Hip (cm) | 103.11 ± 10.36  (n=100) | 107.60 ± 18.10  (n=511) | *<0.001* |
| Waist-to-hip ratio | 0.82 ± 0.11  (n=100) | 0.97 ± 0.10  (n=511) | *<0.001* |
| Systolic blood pressure (mmHg) | 126.88 ± 14.85  (n=105) | 138.94 ± 18.73  (n=688) | *<0.001* |
| Diastolic blood pressure (mmHg) | 76.84 ± 10.25  (n=105) | 77.59 ± 13.60  (n=688) | 0.711 |
| Heart rate (bpm) | 70.18 ± 10.84  (n=102) | 73.93 ± 10.90  (n=687) | *<0.001* |
| MEDICAL HISTORY | |  |  |
| Diabetes duration (years) | 0.00 ± 0.00  (n=105) | 13.27 ± 9.87  (n=593) | *<0.001* |
| Current alcohol consumption | 28 (26.7%)  (n=105) | 156 (22.8%)  (n=684) | 0.455 |
| Current smoking | 13 (12.5%)  (n=104) | 116 (16.9%)  (n=685) | 0.319 |
| Diet treatment | 3 (3.9%)  (n=76) | 507 (97.5%)  (n=520) | *<0.001* |
| CONCOMITANT DISEASES | |  |  |
| Liver disease | 1 (7.7%)  (n=13) | 75 (19.5%)  (n=385) | 0.481 |
| Endocrine disease | 6 (42.9%)  (n=14) | 94 (24.0%)  (n=392) | 0.195 |
| Heart failure | 3 (25.0%)  (n=12) | 109 (28.3%)  (n=385) | 1.000 |
| Hypertension | 7 (43.8%)  (n=16) | 388 (88.6%)  (n=438) | *<0.001* |
| Hyperlipidaemia | 1 (8.3%)  (n=12) | 270 (69.8%)  (n=387) | *<0.001* |
| Obesity | 4 (30.8%)  (n=13) | 247 (61.1%)  (n=404) | 0.056 |
| LABORATORY PARAMETERS | |  |  |
| White blood cell count (G/L) | 6.48 ± 1.62  (n=38) | 8.28 ± 2.83  (n=498) | *<0.001* |
| Red blood cell count (T/L) | 4.70 ± 0.39  (n=37) | 4.73 ± 0.57  (n=499) | 0.619 |
| Haemoglobin (g/L) | 135.89 ± 11.67  (n=37) | 138.68 ± 18.83  (n=499) | 0.180 |
| Haematocrit (%) | 39.77 ± 2.76  (n=26) | 41.30 ± 5.13  (n=435) | *0.039* |
| MCV (fL) | 86.00 ± 3.52  (n=37) | 87.13 ± 7.91  (n=499) | *0.031* |
| MCHC (g/L) | 336.68 ± 10.83  (n=37) | 322.01 ± 65.81  (n=496) | 0.510 |
| Platelet count (G/L) | 260.53 ± 59.37  (n=36) | 256.79 ± 73.88 (n=491) | 0.703 |
| Glucose (mmol/L) | 5.07 ± 0.62  (n=48) | 8.96 ± 4.26  (n=514) | *<0.001* |
| Urea nitrogen (mmol/L) | 4.46 ± 1.10  (n=37) | 6.84 ± 4.92  (n=499) | *<0.001* |
| Creatinine (µmol/L) | 72.03 ± 13.75  (n=37) | 88.68 ± 39.66  (n=501) | *0.004* |
| eGFR (mL/min/1.73 m^2^) | 102.57 ± 15.69  (n=37) | 80.95 ± 25.96  (n=501) | *<0.001* |
| Uric acid (µmol/L) | 250.88 ± 56.30  (n=33) | 290.02 ± 79.93  (n=343) | *0.003* |
| C-reactive protein (mg/L) | 2.40 ± 2.01  (n=24) | 11.44 ± 36.35  (n=278) | *<0.001* |
| Total calcium (mmol/L) | 2.33 ± 0.08  (n=33) | 2.39 ± 0.22  (n=339) | *0.001* |
| Sodium (mmol/L) | 139.14 ± 2.56  (n=35) | 138.88 ± 3.08  (n=509) | 0.733 |
| Potassium (mmol/L) | 4.32 ± 0.38  (n=34) | 4.38 ± 0.52  (n=508) | 0.514 |
| Phosphate (mmol/L) | 1.10 ± 0.17  (n=28) | 1.08 ± 0.23  (n=155) | 0.474 |
| Magnesium (mmol/L) | 0.89 ± 0.05  (n=33) | 0.79 ± 0.12  (n=288) | *<0.001* |
| Total protein (g/L) | 73.03 ± 3.62  (n=32) | 70.34 ± 8.72  (n=326) | 0.035 |
| Albumin (g/L) | 48.00 ± 2.86  (n=34) | 45.65 ± 5.00  (n=398) | *<0.001* |
| Total cholesterol (mmol/L) | 5.08 ± 1.05  (n=46) | 4.78 ± 1.41  (n=353) | *0.039* |
| Triglycerides (mmol/L) | 1.49 ± 0.98  (n=46) | 2.63 ± 5.08  (n=359) | *<0.001* |
| LDL (mmol/L) | 2.42 ± 0.70  (n=34) | 2.49 ± 1.06  (n=312) | 0.988 |
| HDL (mmol/L) | 1.54 ± 0.44  (n=41) | 1.27 ± 0.44  (n=337) | *<0.001* |
| GOT (U/L) | 19.34 ± 7.26  (n=38) | 26.47 ± 17.03  (n=461) | *<0.001* |
| GPT (U/L) | 23.08 ± 22.82  (n=38) | 31.16 ± 20.73  (n=460) | *<0.001* |
| ALP (U/L) | 63.80 ± 20.14  (n=35) | 85.19 ± 33.67  (n=429) | *<0.001* |
| GGT (U/L) | 19.11 ± 19.54  (n=38) | 42.85 ± 70.43  (n=460) | *<0.001* |
| Total bilirubin (µmol/L) | 10.46 ± 7.82  (n=36) | 8.81 ± 4.87  (n=424) | 0.468 |
| Creatine kinase (U/L) | 92.80 ± 43.83  (n=20) | 112.92 ± 77.57  (n=145) | 0.356 |
| HbA1c (%) | 5.44 ± 0.69  (n=45) | 8.50 ± 5.53  (n=355) | *<0.001* |
| TSH (mIU/L) | 3.08 ± 4.34  (n=29) | 3.15 ± 12.23  (n=317) | 0.069 |
| Urine pH | 6.11 ± 0.56  (n=23) | 5.65 ± 0.70  (n=339) | *<0.001* |
| CARTs |  |  |  |
| HR response to deep breathing (beats/min) | 25.33 ± 8.15  (n=105) | 15.12 ± 11.28  (n=692) | *<0.001* |
| Valsalva ratio | 1.75 ± 0.46  (n=105) | 1.36 ± 0.86  (n=686) | *<0.001* |
| 30/15 ratio | 1.15 ± 0.12  (n=105) | 1.04 ± 0.11  (n=671) | *<0.001* |
| SBP response to standing (mmHg) | 3.84 ± 5.79  (n=105) | 8.02 ± 10.39  (n=671) | *<0.001* |
| DBP response during sustained handgrip (mmHg) | 18.07 ± 4.83  (n=105) | 12.45 ± 10.11  (n=544) | *<0.001* |
| Autonomic score | 0.71 ± 0.90  (n=105) | 3.98 ± 2.06  (n=693) | *<0.001* |

*Abbreviations: ALP, alkaline phosphatase; BMI, body mass index; bpm, beats per minute; CART, cardiovascular autonomic reflex test;* *DBP, diastolic blood pressure; dCAN, definite cardiac autonomic neuropathy; eGFR, estimated glomerular filtration rate; GGT, gamma-glutamyl transferase; GOT, glutamate oxaloacetate transaminase; GPT, glutamate pyruvate transaminase; HbA1c, glycated hemoglobin; HDL, high-density lipoprotein cholesterol; HR, heart rate; LDL, low-density lipoprotein cholesterol;* *MCV, mean corpuscular volume; MCHC, mean corpuscular hemoglobin concentration; Sig., statistical significance; Stat., statistical test; SBP, systolic blood pressure; TSH, thyroid-stimulating hormone; T2DM, type 2 diabetes mellitus.*

*Variables with a normal distribution are presented as mean ± standard deviation (SD), whereas non-normally distributed variables are reported as median (interquartile range [IQR]).* *Italicized p-values indicate statistically significant results.*
